# Study of the effect of excited state concentration on photodegradation of the p3ht polymer

**DOI:** 10.1038/srep33238

**Published:** 2016-09-15

**Authors:** V. N. Peters, Rohan Alexander, D’Angelo A. Peters, M. A. Noginov

**Affiliations:** 1 Center for Materials Research, Norfolk State University, Norfolk, VA 23504, USA; 2 Summer Research Program, Center for Materials Research, Norfolk State University, Norfolk, VA 23504, USA; 3 School of Engineering, University of Michigan, Ann Arbor, MI 48109, USA.

## Abstract

We have studied photoinduced reduction of absorption and emission in p3ht, a semiconducting polymer, and found that the rate of photodegradation (destruction of the constituent thiophene rings) does not correlate with the luminescence intensity and, correspondingly, does not depend on the excited state concentration. This conclusion rules out Purcell enhancement of radiative decay rate as a possible explanation of the recently discovered reduction of the p3ht photodegradation rate in the vicinity of metallic substrates and lamellar metal-dielectric metamaterials.

It is well known that spatially inhomogeneous dielectric environments, which can be as simple as interfaces between the two media or as complex as nanostructured composite materials metamaterials^1^,^2^,^3^, can control scores of physical phenomena, including the rates^4^,^5^,^6^,^7^, the spectra^8^,^9^, and the directionality^10^ of spontaneous emission, stimulated emission^11^, and Förster energy transfer^12^,^13^,^14^. Following the arguments of the Marcus theory^15^,^16^, which states that the rates of chemical reactions (determined by the reorganization (polarization) energies of surrounding molecules) critically depend on *local* dielectric permittivities at optical and microwave frequencies, one can further infer that *nonlocal* dielectric environments, *e.g.* the vicinity to metallic surfaces, can influence the rates of chemical reactions as well.

This hypothesis has been experimentally tested in our recent work^17^, in which we studied the effect of metallic and lamellar metal-dielectric substrates on photodegradation of the poly-3-hexylthiophene (p3ht) semiconducting polymer. (The structure of p3ht, composed of thiophene rings with attached alkyl chains, as well as its absorption and emission spectra are depicted in Fig. 1.) It has been found that under white light (halogen lamp) illumination, there are two known from the literature mechanisms that contribute to photodegradation of p3ht. In the first (radical chain) process, oxygen-based radicals attack the π-carbon atom of the alkyl side chain, by abstraction of hydrogen, leading to chain scission and photobleaching^18^,^19^. The corresponding action spectrum rises toward short, ultraviolet (UV), wavelengths. In the second process, singlet oxygen, whose formation is facilitated by photoexcitation of p3ht, attacks the polymer backbone, leading to photobleaching without influencing the side chains^18^,^20^. In the latter case, the action spectrum of photodegradation follows the absorption spectrum of the polymer^18^. Both mechanisms cause reduction of the p3ht absorption band. At the same time, random scission of the polymer backbone, which is characteristic of the singlet oxygen mechanism, causes formation of a large number of short polymer chains and a corresponding blue shift of the absorption spectrum^21^.

The major experimental result of ref. 17, which is most closely related to the present short communication, is as follows: The rates of photodegradation of the p3ht films deposited onto metallic or layered metal-dielectric substrates (with MgF_2_ spacer on top) are smaller than those in similar p3ht films deposited onto purely dielectric substrates. This result has been interpreted as an experimental evidence of inhibition of a chemical reaction by nonlocal dielectric environment^17^.

At the same time, one question of fundamental and practical importance has *not* been addressed in ref. 17. Thus, photodegradation of organic molecules often stems from a much higher reactivity of excited molecular states than the ground state^22^. Therefore, the vicinity to metallic surfaces and lamellar metal-dielectric metamaterials can shorten the decay time of the p3ht excited state (*via* increased density of photonic states^6^,^23^, leading to enhancement of the Purcell factor^24^) and, correspondingly, affects the rate of the photodegradation.

The purpose of the present work was to study the effect of the p3ht excited state concentration on the rate of its photodegradation and answer the question whether the enhanced density of photonic states and enhanced Purcell factor in the vicinity of metamaterials and metallic surfaces can explain the result of ref. 17 discussed above.

## Model

Let us start with the simple analytical model and consider the rate equations describing the concentrations of the molecular ground state *n*_*0*_ [cm^−3^] and the excited state *n*_*1*_ [cm^−3^]:


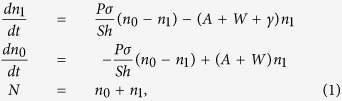


where *P* [W/cm^2^] is the pumping power (assumed to be monochromatic); *S* [cm^2^] is the pumped spot area; σ[cm^2^] is the absorption cross section; *h* [J·s] is the Planck’s constant; *A* [s^−1^] is the radiative decay rate; *W* [s^−1^] is the non-radiative decay rate; *γ* [s^−1^] is the rate of photodegradation, turning molecules (thiophene rings) into the reaction products; and *N* [cm^−3^] is the total concentration of the molecules. Correspondingly, the rate equation describing the overall reduction of *N* due to the chemical reaction reads:


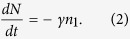


At weak pumping used in our experiment, *n*_*1*_≪*n*_*0*_, *N* and *n*_*0*_ − *n*_*1*_ ≈ *N*. Furthermore, as the characteristic time of photodegradation (hours) is much longer than the excited state decay time (*A *+ *W *+ *γ*)^−1^ (nanoseconds), the time derivative in the first formula of Eq. (1) can be neglected to yield


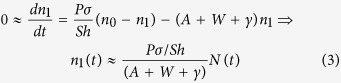


(The second formula of Eq. (3) shows the abovementioned dependence of the excited state concentration *n*_*1*_ on the radiative decay rate *A*). Since the intensity of spontaneous emission *I* [cm^−3.^s^−1^] (the number of photons generated in unit volume per second) is proportional to the excited state concentration *n*_1_,


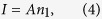


the slow time dependence *n*_*1*_(*t*) (at cw pumping) can be monitored by measuring the time dependence of the spontaneous emission *I*(*t*). Furthermore, by combining Eqs (2) and (4), one gets


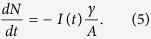


Photoinduced luminescence quenching in p3ht is reportedly governed by several processes occurring at strongly different time scales^21^,^25^. Thus, the collisional quenching (due to encounter of the polymer excited states with the ground state oxygen) is nearly instantaneous; the light-assisted formation of a charge transfer complex between the polymer and oxygen^26^,^27^ occurs within minutes, and reduction of the luminescence intensity due to photo-oxidation of the polymer takes hours^28^. The products of the latter photo-oxidation act as efficient luminescence quenching centers. Correspondingly, a small loss in absorbance has been reported to lead to a substantial photoluminescence quenching^21^,^25^.

Therefore, the experimentally measured emission kinetics *I*(*t*) (at cw pumping) is expected to have a complex character. Substituting it to Eq. (5) and solving the equation numerically, one can obtain the calculated dependence *N*^*calc*^(*t*) (with *γ/A* being a fitting parameter), which can be compared with the experimental dependence *N*^*exp*^(*t*). If the experimental and the calculated curves *N*(*t*) will match each other, then the change of the p3ht photodegradation rate in the vicinity of metal-dielectric substrates^17^ can be explained by the modified density of photonic states and enhanced Purcell enhancement factor. However, if the experimental and the calculated curves *N*(*t*) will not agree with each other, an alternative explanation for the experimental findings of ref. 17 will have to be sought.

### Experimental samples

The material of choice in the present study was the same poly-3-hexylthiophene (p3ht) polymer, which was used in our recent work^17^. The preparation and characterization of the experimental samples – thin p3ht films deposited onto glass substrates – is discussed in Methods.

In order to study the effect of aging on the photodegradation of p3ht, some films were made from fresh solutions (prepared in the same day) while other films were made from the solutions, which were several days or several weeks old. Furthermore, some films were measured in the same day they were prepared, while other films were stored for several days before they were experimentally studied. (All solutions and films were stored in the dark, in an ambient room atmosphere at room temperature.)

### Experimental Measurements

**Figure 2:**
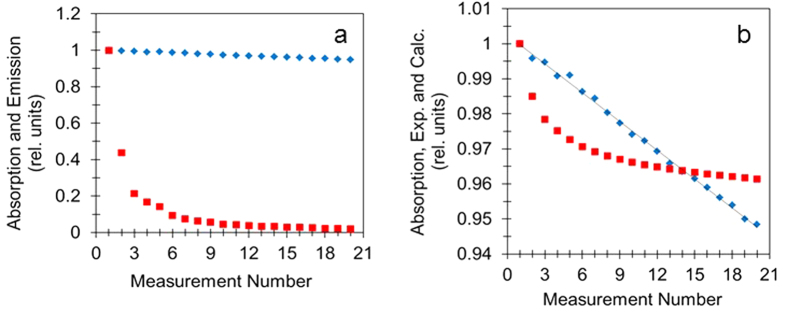
(**a**) Photoinduced reduction of absorption (~*N*^*exp*^(*t*), blue diamonds) and emission (*I*^*exp*^(*t*), red squares) with increased number of photoexposure cycles. (**b**) Comparison of the experimentally measured photodegradation *N*^*exp*^(*t*) (same as in Fig. 2a, blue diamonds) and the one calculated under the assumption that the rate of photodegradation is proportional to the excited state concentration *N*^*calc*^(*t*) (red squares).

In our studies, we performed multiple repetitive photo-exposures and measurements of absorption and emission spectra of p3ht films. The only source of photo-exposure in these experiments was the probing light in the spectrophotometer, which was used to measure absorption, and the excitation light in the spectrofluorimeter, which was used to measure emission. The typical scan parameters are described in Methods. An absorption scan followed by an emission scan composed a *cycle*. The cycle number (termed Measurement Number) is shown on the horizontal axis in Fig. 2a,b, as discussed below.

## Results and Discussion

Photo-exposure of p3ht films caused reduction of their absorption and emission. This is evident from Fig. 2a, depicting absorption and emission maxima plotted versus the number of photo-exposure cycles. The decay of absorption, ~*N*^*exp*^(*t*), (by ≈5% after 20 photo-exposure cycles) was nearly linear. Over the same time, the emission intensity *I*^*exp*^(*t*) decreased nearly twentyfold, and its decay was neither linear nor exponential, Fig. 2a. After substituting the experimental emission decay kinetics to Eq. 5 and performing numerical integration, we have obtained the calculated dependence *N*^*calc*^(*t*), which is compared to the experimental curve *N*^*exp*^(*t*) in Fig. 2b. (Note that the dependence on *t* is equivalent to the dependence on the number of photoexposure cycles.) The constant γ/A was chosen to bring the calculated curve *N*^*calc*^(*t*) to the range of the experimental values *N*^*exp*^(*t*) (it does not affect the shape of the curve).

The film, whose decay kinetics are shown in Fig. 2, was spin-coated using freshly made solution and experimentally studied in the same day. The kinetics in (i) the film made from a month-old solution and measured the same day, Fig. SI-1, and (ii) the film prepared from a fresh solution and measured three days later, Fig. SI-2, are shown in the Supplementary Information section. They are qualitatively similar to the ones in Fig. 2 in a sense that the shapes *N*^*calc*^(*t*) and *N*^*exp*^(*t*) are wholly different. In two out of six samples studied, the absorption decay curves *N*^*exp*^(*t*) were very slightly arched, Fig. SI-2. Yet, the experimental *N*^*exp*^(*t*) and the calculated *N*^*calc*^(*t*) curves, qualitatively similar to those in Fig. 2b, were dramatically different from each other. We did not find any correlation between the behavior of the *N*^*exp*^(*t*) and *N*^*exp*^(*t*) curves and aging of the films or the solutions.

We, thus, have shown that the rate of photodegradation of p3ht does not depend on the luminescence intensity *I* and excited state concentration *n*_*1*_, contrary to the predictions of Eqs 4 and 5. This rules out Purcell enhancement of spontaneous emission as a possible explanation of the reduced photodegradation rate in the vicinity of metallic surfaces and lamellar hyperbolic metamaterials reported in our recent study^17^. This brings us back to the line of reasoning of ref. 17, suggesting that the explanation of the observed phenomenon should be sought in the arguments of the Marcus theory.

## Summary

We have experimentally studied photo-induced reduction of absorption and emission in the p3ht semiconducting polymer. We have found that the rate of photodegradation of the polymer (destruction of thiophene rings and reduction of absorption) does not correlate with the luminescence intensity and, correspondingly, does not increase proportionally with the increase of the excited state concentration. This conclusion is seemingly surprising, because one of the two known mechanisms of p3ht degradation – singlet oxygen mechanism – is predicted to depend on the excited state concentration of the thiophene rings constituting the polymer^25^. At the same time, the efficiency of the singlet oxygen photodegradation mechanism in solid p3ht films is reportedly small in comparison with that of the UV light-triggered radical chain mechanism^18^,^29^, making the conclusion above plausible.

The experimental results of this study lead us to the conclusion of practical importance – that reduced photodegradation of p3ht in vicinity of metallic surfaces and lamellar hyperbolic metamaterials^17^ cannot be explained by the increased density of photonic states and the Purcell enhancement of the spontaneous emission decay rate^24^,^30^. Instead, as discussed below, the explanation for the latter phenomenon is to be sought in the arguments of the Marcus theory.

Recently, the reduction of both charge separation and charge recombination rates in the triphenylene: perylene diimide dyad {TriPh(donor): PerDi(acceptor)} has been observed in vicinity of metallic surfaces and lamellar metal-dielectric metamaterials^31^. The qualitatively similar effect (reduction of the recombination rate) has been reported in the p3ht:PC_60_BM polymer blend^31^. These two experiments show the generality of our recent finding^17^ (reduction of the rate of a chemical reaction in vicinity of metal). The results of ref. 31 have been explained in terms of the modified Marcus theory. In particular, it has been shown that interaction of molecular dipoles with their mirror images reduces the dipole energy the same way as modification of the local dielectric permittivity does, leading to the reduction of the reaction rates. We infer that the same explanation is applicable to the experimental result of our ref. 17 (re photodegradation of p3ht).

## Methods

### Experimental Samples

The material of choice in the present study was the same poly-3-hexylthiophene (p3ht) polymer (98% head to tail regioregular, M_n_ = 54,000–75000, from Sigma Aldrich), which was used in our recent work^17^. The polymer was dissolved in chloroform (ACS grade, from Fischer Scientific) to a concentration of 10–12 mg/ml. It was spin coated onto glass substrates at 500, 3000, then 7000 rpm for a total of 25s (using the Spin coater P6700 from Specialty Coating Systems) and dried in air. The thickness of the fabricated thin films (measured with the Bruker DekTakXT profilometer) ranged from 65 nm to 105 nm, depending on the concentration of p3ht in the solution.

### Experimental Scan Parameters

The only source of photo-exposure in multiple repetitive measurements of absorption and emission spectra of p3ht films was the probing light in the spectrophotometer (Perkin Elmer Lambda 900) and the excitation light in the spectrofluorimeter (Horiba Scientific Fluorolog 3). The typical scan parameters were as follows. (i) Absorption measurements. Scan range: 320 nm–800 nm, increment: 2 nm, bandwidth (slit): 2 nm, integration time: 1.52 s, scan time: 6 min. (ii) Emission measurements. Excitation wavelength: 520 nm (absorption maximum of p3ht^20^), excitation bandwidth (slit): 14.7 nm, scan range: 560 nm–800 nm, increment: 5 nm, integration time: 10 s, scan time: 8 min. Approximately 30 seconds between the scans was needed for removing the sample from one instrument and setting it up for next the scan in the other instrument.

## Additional Information

**How to cite this article**: Peters, V. N. *et al*. Study of the effect of excited state concentration on photodegradation of the p3ht polymer. *Sci. Rep.* **6**, 33238; doi: 10.1038/srep33238 (2016).

## Supplementary Material

Supplementary Information

## Figures and Tables

**Figure 1:**
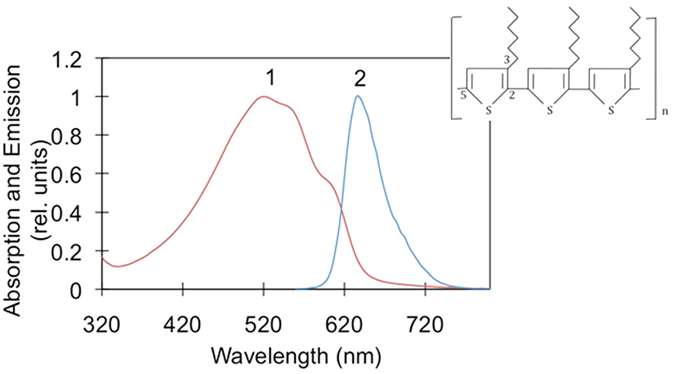
Absorption (1) and emission (2) spectra of p3ht polymer. Inset: polymer’s structure.
