# Supplementary material for: Study of the effect of excited state concentration on photodegradation of the p3ht polymer
Source: Sci Rep. 2016 Sep 15;6:33238. doi: 10.1038/srep33238 (PMC5024085; doi:10.1038/srep33238)
Supplement: Supplementary Information [file srep33238-s1.pdf]

Study of the effect of excited state concentration on photodegradation of the p3ht polymer

V. N. Peters[1], Rohan Alexander[2,3], D'Angelo A. Peters[2], M. A. Noginov[1,*]

[1] *Center for Materials Research, Norfolk State University, Norfolk, VA 23504*

[2] *Summer Research Program, Center for Materials Research, Norfolk State University, Norfolk, VA 23504*

[3] *School of Engineering, University of Michigan, Ann Arbor, MI 48109*

**Corresponding author: mnoginov@nsu.edu*

Supplementary Information

The film, whose decay kinetics are shown in Fig. 2 of the main text, was spin-coated using freshly made solution and experimentally studied in the same day. The kinetics in (i) the film made from a month-old solution and measured the same day and (ii) the film prepared from a fresh solution and measured three days later are shown below (Figs. SI-1 and SI-2, correspondingly). They are qualitatively similar to the ones in Fig. 2, in a sense that the shapes $N^{calc}(t)$ and $N^{exp}(t)$ are wholly different. We did not find any correlation between the behavior of the $N^{exp}(t)$ and $N^{exp}(t)$ curves and aging of the films or the solutions.

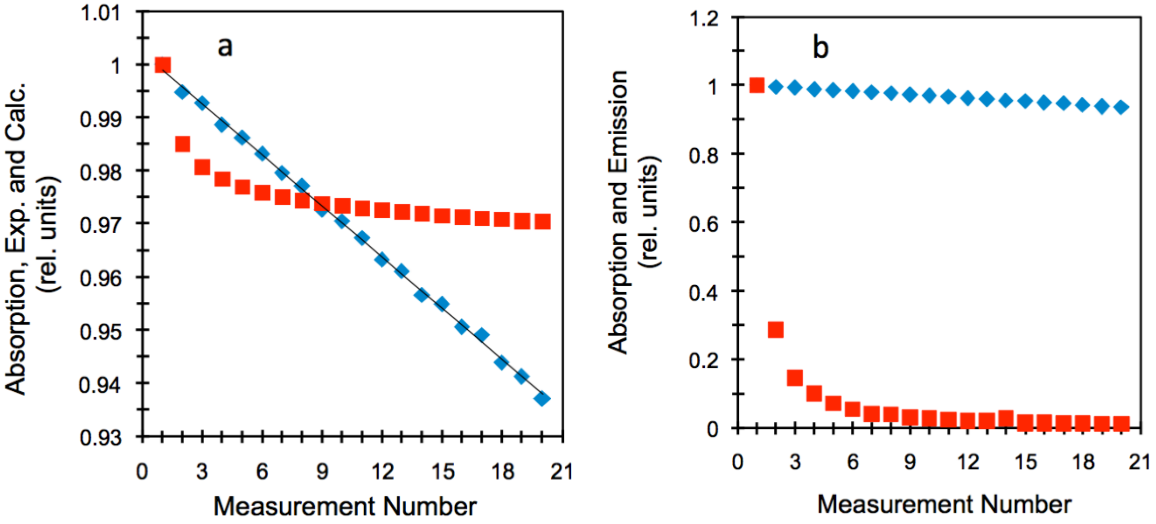

Fig. SI-1. (a) Photoinduced reduction of absorption (~$N^{exp}(t)$, blue diamonds) and emission ($I^{exp}(t)$, red squares) with increased number of photoexposure cycles in the film made from a month-old solution and measured in the same day. (b) Comparison of the experimentally measured $N^{exp}(t)$ (blue diamonds) and calculated $N^{calc}(t)$ photodegradation (red squares). See the main text for calculation of $N^{calc}(t)$.

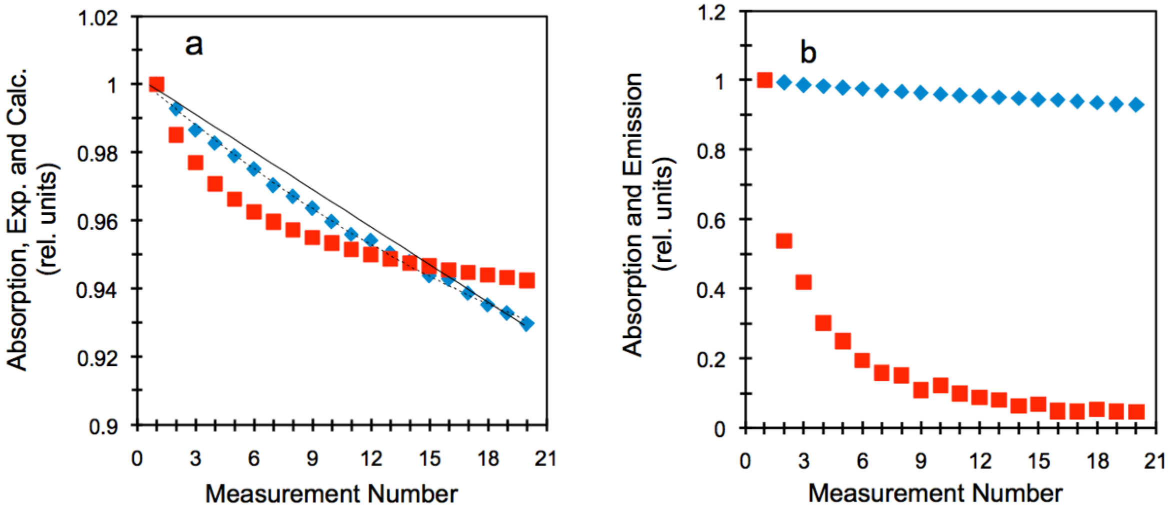

Fig. SI-2. Same as Fig. SI-1 for the film prepared from a fresh solution and measured three days later. Dashed solid line shows slight arching of the $N^{exp}(t)$ curve; black solid line is the straight line.
